# Lessons from Ethiopian pharmaceutical regulation: a risk-based approach to overcome challenges and unlock opportunities

**DOI:** 10.3389/fmed.2025.1484649

**Published:** 2025-09-04

**Authors:** Hassen Kebede Hassen, Yesuneh Tefera Mekasha, Addisu Afrassa Tegegne, Yildiz Ozalp

**Affiliations:** ^1^Veterinary Drug Quality and Standards Regulatory Unit, Ethiopian Agricultural Authority, Addis Ababa, Ethiopia; ^2^Department of Veterinary Pharmacy, Pharmaceutical Quality Assurance and Regulatory Affairs, University of Gondar, Gondar, Ethiopia; ^3^Department of Pharmaceutical Chemistry, School of Pharmacy, College of Medicine and Health Sciences, University of Gondar, Gondar, Ethiopia; ^4^Department of Pharmaceutical Technology, Faculty of Pharmacy, Near East University, Nicosia, Türkiye

**Keywords:** postmarket surveillance, regulatory constraints, regulatory risk, poor quality, supply chain, pharmaceutical products, Ethiopia

## Abstract

**Background:**

Pharmaceutical products are essential for disease prevention, treatment, and public health promotion. With the rapid growth of the global pharmaceutical industry in terms of bulk and variety, ensuring the safety and efficacy becomes critical. However, in resource-limited settings like Ethiopia, problems with the regulatory system and a lack of updated quality information hamper access to quality medicines.

**Objectives:**

This study, conducted from September 2021 to December 2023, aimed to examine the challenges and opportunities within Ethiopia’s pharmaceutical regulation, using retrospective data and analyzing current regulatory perspectives on human medicinal products.

**Methods:**

Retrospective data from published online databases, such as Google Scholar, PubMed, and Web of Science, are searched using specific keywords. Cross-sectional regulatory analysis undertaken through a focus group discussion and questionnaire survey on regulatory experts and stakeholders from the supply chain. The data were organized, filtered for relevance, and analyzed descriptively, with findings presented through tables, flowcharts, and contextual narratives.

**Results:**

The retrospective data revealed that 21.4% of product samples taken from the market were found to be of poor quality. Cross-sectional regulatory analysis indicated constraints within the current supply chain, such as suboptimal supply volumes (36.17%), insufficient variety of medicines (55.32%), issues related to foreign currency (65.96%), the presence of varying degree of corruption at any one of the segments in regulatory system (85%), and dependency on previous brands (27.7%) have been indicated. From focus group discussions with regulatory experts, it is evident that the regulatory authority (Ethiopian Food and Drug Authority (EFDA)) faces problems with application backlogs. From expert opinion-based analysis on regulatory risk, suboptimal performance (risk priority number (RPN) = 75), lack of transparency and consistency (RPN = 64), and problems in traceability and documentation are identified as primary risk factors contributing to regulatory failure. Preparation of guidelines for all activities, adherence to established policies, standard protocols, service timing standard operating procedures (SOPs), and the use of online process monitoring schemes were indicated as the most effective mechanisms to manage the likelihood of regulatory failures.

**Conclusion:**

The challenges within the regulatory processes are reflected by the presence of poor-quality products in post-marketing study findings, deficiencies in regulatory enforcement, and services indicated during interactive assessment. The pharmaceutical supply chain faces challenges that could impact the safety and efficacy of medicines. The findings thus suggest the need to design the system based on perceived risk analysis and improve the regulatory infrastructure that can better mitigate quality and safety concerns.

## Introduction

The regulatory framework in Ethiopia initially operated under the “Pharmacists and Druggists Proclamation No. 43/1942,” regulating both professionals and their facilities. Comprehensive pharmaceutical regulation began with the enactment of “Pharmacy Regulation No. 288/1964,” establishing the legal foundation for drug regulation. Until June 1999, the Pharmacy and Laboratory Department under the Ministry of Health handled medicine regulation, which shifted with the “Drug Administration and Control Proclamation No. 176/1999,” leading to the establishment of the Drug Administration and Control Authority (DACA) in 2001 (1). In 2010, DACA was restructured into the Food, Medicine, and Health Care Administration and Control Authority (EFMHACA) under “Proclamation No. 661/2009,” with expanded responsibilities that included food and health care regulation (2). Currently, human medicinal products in Ethiopia are regulated by the Ethiopian Food and Medicine Authority under Proclamation No. 1112/2019. Ethiopia is currently working on implementing Good Manufacturing Practices (GMP) and regulatory standards in the pharmaceutical industry. The Ethiopian Food and Medicine Authority, under Proclamation No. 1112/2019, Article 20, Subarticle 4, mandates that all medicines and medical devices must be manufactured in compliance with GMP (3). This effort aims to enhance access to safe, high-quality, and effective medicines by streamlining the market authorization process. However, there are issues with the regulatory system implementation.

Pharmaceutical products, also known as medicines or drugs, are specially prepared substances used in modern medicine that are essential for the prevention and treatment of diseases and for the protection of public health (4). These products are formulated to have therapeutic effects and are used to diagnose, cure, mitigate, treat, or prevent various health conditions (5). They come in various forms, such as tablets, capsules, injections, creams, and liquids, and can be prescribed by healthcare professionals or purchased over-the-counter for self-medication. The development, production, and regulation of pharmaceutical products are critical to ensuring their safety, efficacy, and quality in safeguarding public health (6). The issue of poor-quality medicines in Africa, including Ethiopia, is increasingly recognized as a critical public health concern, with its root cause linked to inadequate regulatory frameworks and ineffective policy implementation (7). In Ethiopia, weak regulatory enforcement, as described by findings in the continued presence of illegal medicine outlets, was reported in 2016 (8). A study from Western Ethiopia, around Jimma, revealed that the compliance rate was only 54.76% against the regulatory standards issued (9). More recently, Mekasha et al. (10) have reported a lack of regulatory compliance issues in Ethiopian medicine retail outlets, with drug samples from non-compliant outlets failing to meet quality standards, all these indications resultant distribution of poor-quality medicines.

Distribution of substandard and poor-quality medicines, undermining public health and the credibility of the pharmaceutical sector (8). The widespread occurrence of low-quality pharmaceuticals in Ethiopia (11) presents considerable challenges for the nation’s healthcare institutions, adversely affecting patient safety, the effectiveness of treatments, and the overall state of public health. A study by Sultan et al. indicates that Ethiopia experiences a notable prevalence of substandard mebendazole, albendazole, and tinidazole (12). The prevalence of defective products in the Ethiopian pharmaceutical market is largely rooted in legal and regulatory shortcomings within the sector (8). This may limit resources as inadequate infrastructure and a lack of trained personnel hamper effective post-market surveillance (PMS). The Ethiopian Food and Drug Authority (EFDA) has made progress in improving PMS and regulatory oversight; however, challenges, such as the presence of poor-quality medicines (11, 13) and inadequate regulatory enforcement, remain within the system (8).

Building a robust regulatory framework, based on the adoption of quality by design principles within the regulatory organ, has now received attention for reducing the entry of defective products into the healthcare system (14). Quality by Design plays a crucial role in implementing the International Council for Harmonization (ICH) Q8 and Q9 guidelines. According to the U.S. Food and Drug Administration (FDA), Quality by Design (QbD) is defined as a systematic methodology for the design and development of products and processes (15). This concept was officially recognized by the FDA in 2004, with a comprehensive explanation provided in the document titled “Pharmaceutical cGMPs for the 21st Century: A risk-based Approach.” Historical evidence has shown that the manufacturing of biotechnology products entails numerous intricate steps, which necessitate the management of various quality attributes. These challenges have been effectively addressed through the principles of Quality by Design (16). Growing interest in fulfilling unmet needs in the regulatory setting has led to an increasing focus on assessing various risk-based models and methods. These models aim to enhance the effectiveness and efficiency of regulatory processes, particularly in the pharmaceutical and veterinary sectors (17). Ethiopian regulatory authorities have planned to prioritize resources and attention on high-risk products to ensure that critical medications receive timely and thorough evaluation. Although full implementation of this initiative is still pending, the approach reflects a strategic shift toward a more efficient and risk-based regulatory framework (18).

The concept of the World Health Organization (WHO) Global Benchmarking Tool for regulatory maturity levels is an essential framework used by countries to assess and enhance their national regulatory systems for medicines and vaccines (19). The maturity levels range from 1 to 4, where level 1 indicates the presence of some regulatory functions, and level 4 represents a system that is well-functioning, integrated into the global regulatory environment, and capable of continuous improvement (20). The Ethiopian Food and Drug Authority (EFDA) has made significant strides in enhancing its regulatory framework by aligning its operations with the WHO’s maturity level standards. The EFDA’s initiative to achieve maturity level 3 (ML3) reflects a strong commitment to building a regulatory system that is stable, consistent, and capable of protecting public health (21). Maturity level 2 in the regulatory system, characterized by a reactive approach and evolving national regulatory frameworks, can indeed lead to significant challenges in ensuring the quality of pharmaceuticals (21). In Ethiopia, despite the presence of essential regulatory functions, a circulation of poor-quality products and procedural non-compliance persists in the market, highlighting gaps in policy enforcement and quality assurance regulatory practices.

The objective of this study was to draw lessons from the quality of marketed pharmaceutical products and to identify the regulatory challenges that undermine the efficiency of quality assurance systems. The study was guided by four central research questions: (1) What is the status of pharmaceutical product quality in Ethiopia, based on post-marketing surveillance data? (2) What are the significant regulatory and institutional constraints affecting the pharmaceutical supply chain? (3) Which factors most significantly contribute to regulatory failures in ensuring the quality, safety, and efficacy of pharmaceutical products? and (4) What strategies and reforms could enhance the efficiency and effectiveness of Ethiopia’s pharmaceutical regulatory system?

## Materials and methods

### Study settings

The study was conducted from September 2021 to December 2023, through a comprehensive evaluation of Ethiopia’s pharmaceutical regulatory environment, with a specific focus on institutions that regulate human medicinal products.

Ethiopia is a landlocked East African nation with a population of 120 million people and a land area of 1.1 Mha. It is situated between the borders of Kenya to the south, Eritrea to the north, Sudan to the west, and South Sudan to the south (22). Currently, twenty-two local manufacturing plants exist that formulate drug production. From these, nine companies produce complete product formulations of finished pharmaceutical products using raw materials imported from foreign nations. In contrast, one company produces empty gelatin capsules, and the other factories are engaged in medical supply production (23, 24).

### Study participants

Regulatory experts, product wholesalers, importers, manufacturers, and regulated product retail outlets were included in the study based on convenience.

### Study design and approaches

Retrospective and cross-sectional study designs were employed in this analysis. The retrospective component focused on post-marketing product quality studies, with data collected from online databases. Online databases, such as Google Scholar, PubMed, Web of Science Core Collection, LISTA (EBSCO), and regulatory websites, were used to retrieve data on post-marketing product quality study findings and current activities within the regulatory apparatus. Web search was performed using keywords such as medicines, product quality assessment, *in vitro* drug quality evaluation, physicochemical quality, regulatory, and Ethiopia. A retrospective assessment from an online database used for studying the degree of compliance in marketed products to regulatory standards. All published data encountered before the end of the study period were included in the final analysis.

The cross-sectional study examined existing challenges and lessons to be learned from the regulatory framework, incorporating both questionnaire-based surveys and interactive discussions. The questionnaire targeted the perspectives of stakeholders across the pharmaceutical supply chain regarding prevailing challenges. In contrast, the interactive, checklist-based discussions aimed to gather in-depth insights into the evolution, strengths, and weaknesses of regulatory activities, as well as the key risk factors contributing to regulatory failures toward ensuring the quality, safety, and efficacy of products. The institution-based survey focused on identifying strengths and constraints within the regulatory framework.

### Sampling techniques and data collection methods

A mixed approach combining record analysis, stakeholder opinion surveys, and expert-driven interactive discussions was used. Retrospective data on the quality status of marketed products in Ethiopia were retrieved from all online databases encountered that fit into the search criteria. Convenience sampling of stakeholders from the supply chain among regulatory experts, wholesalers, and importers for inclusion in a cross-sectional study. Existing records related to routine activities in the regulatory institutions, such as inspection reports, compliance records, and others, were conducted as per the Ethiopian Food and Drug Authority’s Risk-Based Guidelines for Post-Marketing Quality Surveillance of Medicines in Ethiopia (25).

Risk analysis in this study was conducted using high-quality risk management tools, particularly Failure Mode and Effects Analysis (FMEA), to assess regulatory risk failure. Criticality was determined using the risk priority number (RPN), based on the likelihood of occurrence (O), severity (S), and detectability (D) of failures, as outlined by Bozdag et al. (26), and previously published scientific findings in a peer-reviewed journal (27, 28). The risk priority number (RPN) scores were assigned through an expert consensus process based on the severity and likelihood of potential problems. The risk identification and prioritization have been undertaken using a group of regulatory experts according to established recommendations (29–31).

Descriptive statistics were used to quantify responses, issues, or conditions. Tables are used to present numerical data, while flowcharts visually represent the variable processes involved in medicine registration and the cause-and-effect of regulatory risk. Narrated texts were used to contextualize the data, providing insights and highlighting key points to make the information more manageable.

## Results and discussion

### Findings from pharmaceutical supply chain: a survey of importers and wholesalers in Ethiopia

In a study evaluating the institutional and supply-related constraints faced by importers and wholesalers within a regulatory system, data were collected. According to the survey responses, 36.17% of participants identified suboptimal current supply volume as a critical challenge that hinders the ability to fulfill demand requirements. This imbalance has been indicated elsewhere as a factor leading to potential shortages and disruptions in the availability of medicinal products (25). In the African context, several limitations significantly impact access to basic medicines, contributing to the poor health metrics observed in many countries across the region (32).

Additionally, an even higher percentage, 55.32% of participants, expressed concern that the current medicinal variety (type of medicines) is insufficient to meet market needs. This indicates a significant gap between supply and demand, which could have profound implications for healthcare delivery. The study identified foreign currency issues (65.96%) as a major constraint and obstacle to maintaining adequate supply volumes. This problem was reported as a critical barrier by a significant portion of the participants. Currently, based on expert discussions, the Ethiopian government has taken measures to manage foreign currency problems for suppliers, merchants, and the private sector by prioritizing and facilitating access to foreign currency needed for medicinal product purposes. This approach aims to address some of the critical challenges in the pharmaceutical and broader import sectors, where access to foreign currency is essential for procuring goods from international markets.

In regions where foreign currency is scarce or subject to significant fluctuation, the cost of importing pharmaceutical products can rise dramatically, further exacerbating supply chain challenges. In developed countries, drug purchasing costs are a significant component of healthcare spending, often consuming a substantial portion of the budget allocated to non-personal healthcare expenses. This can range from 50 to 90% of these costs, highlighting the financial burden that pharmaceuticals place on healthcare systems (33). Managing these costs effectively is crucial for the sustainability of healthcare systems and ensuring that patients have access to the treatments they need. This requires a combination of strategic policy interventions, cost–control measures, and a focus on value-based healthcare delivery. The remaining constraints in regulated product supply problems, as identified through responses to a questionnaire survey from suppliers, are summarized in Table 1.

**Table 1:** Constraints in regulated product supply problems as indicated by suppliers in the questionnaire survey.

Problem indicated	Frequency (*N* = 47)	Frequency	Percentage
Belief in the current supply volume	Surplus	5	10.63
	Comparatively enough	10	21.27
	Suboptimal	17	36.17
	Insufficient	15	31.91
Belief in the current supply by type needed	Surplus	0	
	Comparatively enough	3	6.38
	Suboptimal	18	38.29
	Insufficient	26	55.32
Problems as obstacles to supply volume	Financial capacity	5	10.64
	Foreign currency problem	31	65.96
	shortage of manufacturers	-	
	Absence of demand in the domestic market due to the knowledge and practice	6	12.76
	regulatory procedure problems	5	10.64
Problems as obstacles for the supply type	Financial capacity	-	
	Foreign currency problem	17	36.17
	shortage of manufacturers	2	4.26
	Absence of demand in the domestic market due to the knowledge and practice	16	34.04
	Regulatory procedures	12	25.53
Belief on regulatory institutions to ensure quality and safety of medicines	Excellent	9	19.14
	Very Good	12	25.53
	Good	13	27.65
	No (negative role)	13	27.66

### Findings within pharmaceutical supply chain: regulatory environment

The assessment revealed hurdles that stakeholders experience within the regulatory environment, which can reduce efficiency in the overall pharmaceutical supply system. As shown in Table 2, 85.1% of respondents reported varying degrees of corruption in different segments of the regulatory system, which can hinder the effectiveness of regulations, compromise product quality, and create barriers to market entry for new suppliers.

**Table 2:** Regulatory constraints as indicated by suppliers from the questionnaire survey.

Problems indicated	Frequency	Percentage
A varying degree of corruption	40	85.1
Multiple local agent system	8	17
Dependence on existing brands	13	27.7
Protracted market authorization processes	5	10.6
Non-relevant data inquiry for registration	2	4.2
Non-compliance with the initial registration agreement	1	2.1
Prevalence of malpractices in the supply chain	2	4.2
Lack of accountability for defective product recalls by manufacturers	5	10.6

The study also revealed that 27.7% of respondents view dependency on existing medicinal brands as a significant supply constraint. This dependency can arise from several factors, including the market dominance of certain brands, regulatory barriers that make it difficult for new entrants to compete, and a lack of incentives for innovation and diversification within the market (34). The study’s observations indicated a need to create transparency and encourage market diversity through appropriate guidelines.

### Substandard medicines in the Ethiopian pharmaceutical market: failure rates and implications

In recent retrospective quality assessments of various pharmaceutical products, detailed data were documented by drug name, strength, pharmacological class, pharmacopeial standards applied, sample pass/fail rates, defective parameters, and country of origin of failed products (Table 3). The failed products originated from diverse countries, including Korea, India, Cyprus, Ethiopia, China, and some unspecified sources, indicating that substandard medicines represent a global issue rather than a region-specific problem. Particularly troubling were the multiple failures found among widely used drugs such as paracetamol and ciprofloxacin, which serve as frontline treatments in many healthcare settings.

**Table 3:** General description of retrospective drug quality study findings and analysis.

Drug name and strength	Class of drugs	Standard pharmacopeia	Product sample passed (tablets/capsules)	Product sample failed (tablets/capsules) and had defective parameters	Country of origin for failed products	Reference
Chlorpromazine tablets (100 and 25 mg)	Antipsychotic drugs	BP (2000)	3	1 (disintegration time and dissolution rate)	Korea	(49)
Thioridazine (100 and 25 mg)			2	-	NA	
Artemether-Lum tablet (20/120 mg)	Antimalarial drugs	USP (2007) and BP (2009)	240	-	NA	(50)
ART/LUM tablets (20 mg ART/120 mg LUM)		International Pharmacopeias	73	1 (chemical assay based on Ph. Int.) specification	Unspecified	(51)
Chloroquine (250 mg) and quinine (300 mg) tablets		USP standard specifications and procedures	-	60 (visual inspection, hardness, and weight variation tests)	India/Leben, Cyprus/Remedica	(52)
Pantoprazole tablets (20 mg)	Proton pump inhibitors	USP/NF (2013)	5	-	NA	(53)
Methyldopa (250 mg)	Antihypertensive drugs	USP XXVII, BP (2001)	4	1 (methyl dopa) chemical Assay	Methyldopa, Cyprus	(54)
Furosemide (40 mg)						
				2 (Furosemide) in dissolution test	Furosemide Epharm (Ethiopia)	
Propranolol (40 mg)						
				1 (Propranolol in the identity and hardness test)	Propranolol, India	
Furosemide (40 mg)	Antihypertensive	BP (2009) and USP (2015)	4	_	NA	(55)
Nifedipine tablets (20 mg)	Calcium channel blocking agent	USP/NF (2013).	4	2 (disintegration and dissolution)	Egypt	(56)
Metronidazole tablets (500 and 250 mg)	Antiprotozoal drugs	USP/BP/IP	3	1 (dissolution and disintegration based on BP and USP)	Hindia generic products (metrogyl)	(57)
Albendazole 40 mg per tablet	Antihelminthic drugs	EP, USP	1	1 (dissolution profile)	Bendex, India, CIPLA Ltd., batch no: x21253	(58)
ALB 400 mg		EP (2014a, 2014b, and 2014c), USP (2015a, 2015b, and 2015c)	6	1 ALB (dissolution specification limit based on USP (2015a,c))	Unspecified	(59)
MEB 500 mg				-		
PZQ 40 mg						
				2 PZQ		
ALB 400 mg, MEB 100 mg, and TNZ 500 mg	Anthelminthic agents and antiprotozoal	General and individual monographs as indicated in S1–S2 supporting information	58	48 (dissolution, friability, assay test, and dosage uniformity)	Unspecified	(12)
Albendazole (300, 600, and 2,500 mg per tablet)	Anthelmintic drugs	USP	6	4 (weight uniformity)	Unspecified	(60)
MEB 100 mg	Anthelminthic agent	BP (2007), USPXXVII specifications	5	1 (dissolution and disintegration)	Unspecified	(61)
Ethambutol HCl (100 mg)	Anti-TB	USP (2015)	6	-	NA	(62)
Erythromycin stearate tablets (500 and 250 mg)	Antibacterial agents	BP (2007) and USP (2007)	3	1 (dissolution and assay test)	Sudan	(63)
Doxycycline capsules and tablets (100 mg)		USP 38	9	1 (friability and hardness)	Italy	(64)
Amoxicillin capsule (500 mg)		USP (2007) and BP (2009)	6	-	NA	(65)
Amoxicillin/clavulanate tablets (625 mg)		BP and USP monographs	5	-	NA	(1)
Norfloxacin tablets (400 mg)		USP (2015)	7	2 (dissolution test (USP))	India, South Korea	(66)
Ciprofloxacin tablet (500 mg)		BP (2004), USP/NF	5	1 (dissolution test)	Ethiopia (Addis. Ph. Factory S. C)	(67)
Ciprofloxacin HCl (500 mg)		USP (2008)	6	-	NA	(68)
Ciprofloxacin tablets (500 mg)		BP (2004), USP/NF	7	1 (in uniformity of weight)	Flamingo Pharmaceuticals Ltd., India	(69)
Co-trimoxazole tablets (480 and 960 mg)		BP and USP	6	-	NA	(70)
Metformin hydrochloride tablet (500 mg)	Anti-diabetics drugs	USP, BP	7	-	NA	(71)
Metformin (500 mg)		USP (2015)	5	1 (weight uniformity)	Ethiopia	(72)
Glibenclamide tablets (5 mg)		USP (2007) and BP (2009)	5	-	NA	(73)
Metformin HCl tablets (500 mg)		USP/NF 25, (2007).	5	1 (dissolution profile)	Denk Pharma (Germany)	(74)
Paracetamol tablets (500 mg)	NSAIDs	USP and BP	8	3 paracetamols (assay and disintegration test according to BP)	Ethiopia	(75)
Paracetamol (500 mg)		BP and USP	4	_	NA	(76)
Paracetamol (500 mg)		BP (2001) and USP 23	-	2 (friability),1 (disintegration), and 6 (assay) with sampled 6 brands	China’s	(77)
Ibuprofen tablets (400 mg)		BP, USP	5	1 (brands in disintegration BP)	Unspecified	(78)
Diclofenac sodium tablets (50 mg)	NSAIDs	USP (2007)	6	-	NA	(79)

BP, British Pharmacopeia; USP, United States Pharmacopeia; NA, not available; NSAIDs: nonsteroidal anti-inflammatory drugs; Ph. Int., International Pharmacopeia; Addis. Ph. Factory S. C., Addis Pharmaceutical Factory SC.

The study found that out of 679 samples tested across drug classes, 21.4% failed to meet physicochemical quality standards, highlighting a substantial presence of substandard medicines in the market (Table 3). Antihelminthic drugs exhibited the highest failure rate of 42.9%, indicating difficulties in ensuring uniformity, dissolution, and assay, which could severely compromise treatment efficacy for parasitic infections.

Other drug classes with notable failure rates included NSAIDs (30.3%), antihypertensives (33.3%), and calcium-channel blockers (33.3%), illustrating that quality problems also impact medications for chronic conditions. Despite the largest sample size (374), antimalarial drugs had a 16.3% failure rate, mainly due to defects in assay, hardness, and weight variation, posing an alarming public health risk in malaria–endemic areas. Antiprotozoal and antipsychotic drugs showed failure rates of 25 and 16.7%, respectively, emphasizing the need for rigorous quality control in these categories. Proton pump inhibitors were the only drug class with no reported failures, possibly reflecting higher manufacturing standards or the smaller sample size tested.

Existing evidence from East African countries indicates that 22.6% (151/669) of antimicrobial samples failed at least one quality test. The prevalence of quality failures varied by drug class: 17% in antibiotics (73/432), 24% in antimalarials (41/171), and 56% in anthelmintics (37/66). Among the quality control parameters, the active pharmaceutical ingredient (API) content was the most frequently examined and featured in 93% (14/15) of the included studies (11).

In many low- and middle-income countries (LMICs), national regulatory authorities (NRAs) remain under-resourced. While the World Health Organization’s Global Benchmarking of Regulatory Systems has the potential to be a transformative tool for guiding regulatory strengthening, significant investments are still required to achieve adequate regulatory maturity across all regions (35). The combination of weak regulatory systems, limited resources, inadequate infrastructure, and a lack of trained personnel contributes to the substantial burden of these issues in LMICs (36, 37) (Table 4).

**Table 4:** Retrospective quality assessment based on anatomical therapeutic classification.

Class of drugs	Total sample assessed	Sample with deviated quality parameters	Percentage	Physicochemical quality parameters failed
Antipsychotic drugs	6	1	16.7	Disintegration and dissolution test
Antimalarial drugs	374	61	16.3	Assay of API, hardness, and weight variation
Anti-bacterial drugs, including anti-TB drugs	70	7	10	Dissolution, assay, weight uniformity, disintegration, friability, and hardness
Proton-pump inhibitors	5	-		-
Antidiabetic drugs	24	2	8.3	Dissolution test
Antihelminthic drugs	133	57	42.85	Weight uniformity, dissolution test, friability, assay test, and disintegration test
Antiprotozoal drugs	4	1	25	Disintegration, dissolution, assay test, and friability test
NSAIDs	33	10	30.3	Assay, disintegration, and friability
Antihypertensive’s drugs	12	4	33.3	Dissolution test and weight uniformity
Calcium-channel blocking drugs	6	2	33.3	Disintegration and dissolution
Total	679	145	21.4	

### Evolution of medicinal product regulation in Ethiopia: history, structure, and regional harmonization

Discussions with experts revealed human medicinal products as probably the earliest of all product regulatory schemes in the country. Regulatory evolution in the health sector traces back to the 1940s, when a code of ethics was adopted for pharmacy practice.

The first separately managed medicines regulatory authority was established under Proclamation Number 176/1999 during the establishment of the Drug Administration and Control Authority (DACA), which regulates all agricultural pesticides, veterinary supplies, and human health products. Later on, ceding regulatory roles for pesticides and veterinary supplies to the Ministry of Agriculture and incorporating health service regulation under Proclamation No. 661/2009 for the establishment of the Food, Medicine, and Healthcare Administration and Control Authority of Ethiopia. This proclamation repealed both Proclamation Number 176/1999 for DACA establishment and Public Health Proclamation Number 200/2000 for public health safety proclamation for food, public health hazard substance management, and veterinary drug use issues (1).

Even this ambitious establishment was again revised through Proclamation Number 1112/2019, which established the Ethiopian Food and Medicine Administration, commonly referred to as EFDA. In conclusion, based on its history of development, continuous reform at short intervals seems premature management compared to other countries’ experiences. Currently, the Ethiopian Food and Drug Administration is entitled to regulate medicinal products for human use, cosmetics, tobacco products, medical devices, and *in vitro* diagnostics. In its duty engagement, it is known for market authorization and product licensing, post-marketing surveillance, sample laboratory analysis, clinical trial authorization, advertising regulation, and price regulation (proclaimed but not yet supported with guidelines for implementation). It has direct scope to regulate target institutions engaged in manufacturing, import/export, and wholesale of medicines, medical devices, cosmetics, and tobacco products. Besides medicines, medical devices, cosmetics, and tobacco product regulation, known for collateral finished food product manufacturing, import/export, and wholesale. It is known to operate under the administrative structure of the Ministry of Health, with an optimal level of autonomy in exercising power related to product regulation. The current organogram features three deputy director generals for food, medicines, and medical devices, all reporting to a single director general. Pharmacovigilance and clinical trial lead executive office under the medicines deputy directorate, and this section is responsible for post-marketing surveillance activities.

Medicinal product and medical device registration core process comprises the customer service team, medicinal product registration and licensing, and medical device registration and licensing teams, containing ten-, eighteen-, and five-membered experts, respectively. The customer service team was responsible for collecting applications, screening for modular content, providing corrective feedback, and submitting the application module for review. By profession, all customer service teams are typically staffed with pharmacists. The medicinal product registration and licensing team comprises positions for pharmacists with or without specialization, as well as general medical practitioners with or without specialty. However, currently the team of experts includes pharmacists, pharmacists with a specialty in pharmacology, clinical pharmacy, regulatory affairs, and pharmaceutical analysis. The medical device registration and licensing team invites applications for generalist physicians, biophysicists, biomedical engineers, and medical laboratory technologists. Practically all except medical practitioners and biophysicists are not available in the current setup.

Regulatory authorities have developed a fee structure for the market authorization and licensing of institutions related to human medicines; however, the application of this structure varies across different situations, such as dossier submission and evaluation fees. Notably, the service fee is waived for emergency use authorization. This discrepancy is not only in Ethiopia, as other African nations, including Kenya, Uganda, and Tanzania, also face challenges in implementing regulatory activities within the dossier evaluation framework (38).

From all budgetary shares, it was briefed that registration and market authorization take 0.7% of the total share allocated for the Ethiopian Food and Drug Authority. The overall medicinal product market authorization procedures follow different models for registration applications. The first model involves market authorization by avoiding duplication of effort through regulatory harmonization, allowing medicinal products to be marketed locally once they have been authorized by one or more recognized agencies elsewhere.

The primary responsibility of the regulatory authority for a product to be imported for local sale is to check and verify the registration status in line with what it has declared in the application and product characteristics (formulation and composition), as well as prescribing information (use and dosage precaution) for local marketing, conforms to that agreed in the reference authorization. This model is at its inception stage, where regional harmonization (East African community regulatory harmonization) for medicines regulation is underway (39). When this plan is implemented, products marketed in neighboring countries will have the probability to be authorized for local sale in Ethiopia with all registration credentials declared in the agreed-upon and recognized regulatory agencies. Currently, no medicines are authorized for local sale through this model. A new approach to regulatory harmonization has been initiated by the East African Community (EAC), resulting in shared regulatory requirements and standards for medical product regulation. This initiative simplifies multistate applications, enabling partner states to conduct joint regulatory activities, thereby enhancing efficiency and collaboration in the region (39). The Ethiopian Food and Drug Authority (EFDA) is the lead agency for the Intergovernmental Authority on Development (IGAD) medicines regulatory harmonization initiative^1^. IGAD member states have developed harmonized requirements for the registration of medicines to ensure the efficacy, quality, and safety of these products. Both the East African Community (EAC) and IGAD are working to improve regulatory activities and prevent the presence of quality issues in medicines across the African region.

There is a promising regulatory endeavor in the Southern African community aimed at enhancing regulatory reviews and Good Manufacturing Practices (GMP) inspections. A significant study, Medicine Registration in the Southern African Development Community: Alignment and Strategies for Moving Forward, highlights the progress. The findings indicate that the registration processes and marketing authorization practices of regulatory authorities in Mozambique, Namibia, South Africa, Tanzania, Zambia, and Zimbabwe vary significantly (40). Funding sources for regulatory agencies in Southern Africa vary widely: Namibia relies entirely on government funding, Mozambique mostly on government support with some external contributions, and South Africa splits funding between government (70%) and fees (30%). In Tanzania, funding comes from government (12%), fees (76%), and other sources (12%), while Zambia relies heavily on fees (95%) and minimal external funding (5%). Zimbabwe’s agency is fully funded by fees. Review fees differ by product category (e.g., new chemical entities, biologicals, generics), and no agency charges are incurred for scientific advice (40).

The second model for market authorization regulatory procedure reduces the process of reviewing scientific supporting data that has been accepted and reviewed elsewhere, but includes an abridged independent review of the product in terms of its use under local conditions. This may include review of pharmaceutical (chemistry, manufacturing and controls (CMC)) data in relation to local climatic conditions, distribution facilities, and risk–benefit assessment for use by the local ethnic population, medical practice/culture, disease pattern, and nutrition. Approval by a recognized agency for local marketing is a prerequisite. This model is practiced under EFDA regulatory practice for medicines from countries classified as having recognized and with stringent regulatory authorities globally. World Health Organization prequalification schemes are also treated under this model. The third model for market authorization regulatory procedure relies on the presence of all required levels of experts (internal and external) and facilities for a whole application review process, including all supporting scientific data (quality, preclinical, and clinical) outlined in the common technical document. All market authorization regulatory procedures in the above two models are expected to be treated and passed through this model.

The article also examined regulatory review models—Type 1, Type 2, Type 3A, and Type 3B—using lessons from Zimbabwe’s regulatory review process (41). These insights were applied to identify challenges and opportunities within a regulatory environment similar to Ethiopia’s, offering valuable strategies for improving efficiency and harmonization. The article highlights three models for scientific regulatory review: Type 1 (Verification Review) relies on prior approvals by two or more competent authorities to validate products against existing specifications. Type 2 (Abridged Review) considers local factors and requires prior registration by at least one competent authority. Type 3 (Full Review) encompasses Type 3A, a comprehensive review of quality, safety, and efficacy for products with a Certificate of Pharmaceutical Product (CPP), and Type 3B, a complete, independent evaluation of products that have not undergone prior regulatory review. These models provide flexible approaches for regulatory authorities (42).

Milestones for incrementing regulatory efficiency in the registration of medicines include a regulatory information management system, such as *i Register*, *i Import*, *i License, and i Inspect* application platforms, for automation of key activities in import, registration, and regulatory inspection procedures. These online open-source applications are advocated for increased transparency, improved efficiency, and workforce management. All are designed to account for timing in account for timing in the management of backlogging. The establishment of the national advisory committee (NDAC), comprising academics and representatives from other federal institutions, to obtain critical and updated opinions, as well as the setup to work with government universities to utilize available expertise in reducing the backlog of registration applications, can be considered.

The discussions also highlighted the limitations in the registration process. There is a restricted number of reviewers, insufficient professional diversity within the review panel (including general practitioners, pharmacists, chemists, and physicists), and inadequate departmental organization, particularly concerning the presence of specialties and subspecialties. Comparable regulatory reports had also reported the challenges associated with medicine registration in Ethiopia (8, 43). These factors can contribute to delays and inefficiencies in the registration process, which, in turn, affect the availability and quality of human medicines. Addressing this issue might involve increasing the number of qualified reviewers, broadening the expertise within review panels, and improving departmentalization to ensure more thorough and specialized evaluations.

**Figure 1:**
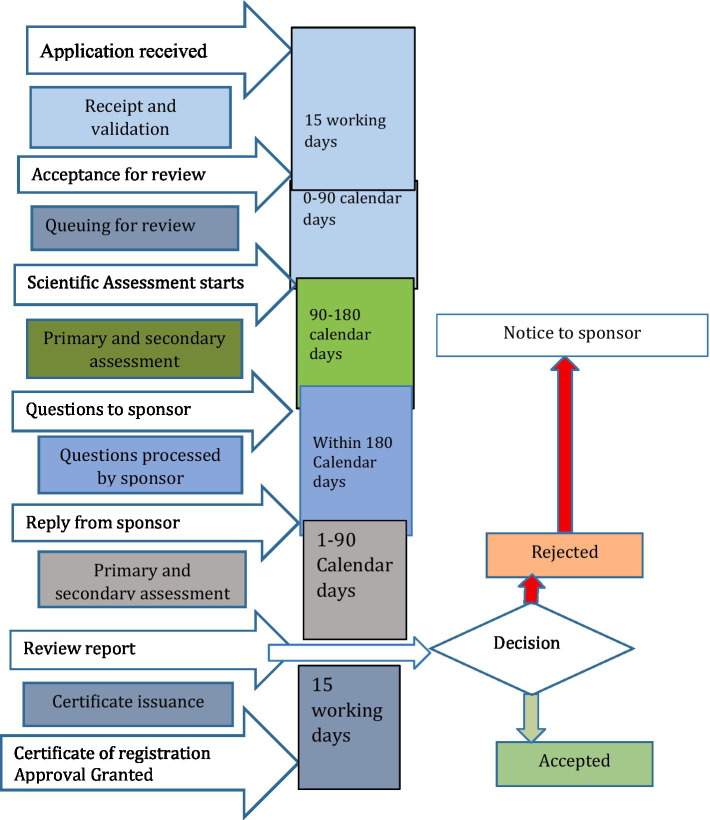
Regulatory framework map for medicine registration by EFDA.

There was also a strong belief in the sharing regulatory assessment reports with stakeholders, with perceived advantages for better experience sharing, building regulatory consistency, and improved access to medicines. However, sharing assessments has also presented hurdles in the form of conflict of interest among manufacturers. The process of routine medicine registration and market authorization in Ethiopia, as derived from group discussions, involves applying along with a product dossier. This application undergoes an audit and review, which is followed or preceded by a Good Manufacturing Practice (GMP) inspection report, culminating in a final verification of the laboratory sample’s quality. Upon successful verification of the sample quality tests, a certificate of market authorization is issued. The regulatory framework map for medicine registration by EFDA was summarized in Figure 1.

### Human resource development and operational processes regulatory quality test center

Human resource recruitment, training, and routine work setup have been discussed with the objective of assessing problems with regulatory design. Human resource recruitment starts with establishing the criteria for selecting applicants. Cornerstones, such as academic background and university academic performance, were taken into consideration. Bachelor’s degrees in pharmacy and chemistry with or without a specialty are only considered. Independent evaluation of applicants was the normal routine practice by blinding applicant identity information from personnel engaged in document evaluation and technical assessment.

Recruitment was then followed by initial tailored training. Trainings start from simple test procedures to complex procedures. In-house training schemes focus on instrument handling, interpretation of the pharmacopeia, and standard operating procedures. Newly joining staff are allowed to practice together with senior analysts before they are allowed to practice independently for 3 months. Theoretical assessment of the International Standards Organization principles, as well as organizational quality management principles, is also undertaken via examinations and is expected to score greater than 60% before being subjected to specific technical procedures. Continuous assessment for performance proficiency is undertaken. It is checked by test result validation procedures. Trainees are allowed to undertake analysis for known samples and will be tested for the results, which will be compared with those of senior analysts. They are allowed to practice independently when their test results on retention samples exactly match those of the senior analyst (target similarity should be greater than 90%). Specific technical training programs are also arranged with European or Asian institutions. However, these options are sponsor-dependent and are not routinely undertaken for all analysts.

Samples are collected routinely from either external customers, such as the police department, or different sections of the food and drug authority, including for PMS assessment, as part of the registration process, or branch offices for investigative purposes. Quality test requests may also come from other African countries. There are established standard operating procedures for sample reception, processing, and result submission. Sample accompanying information is assessed according to standard procedure. Primary standard submission is a mandatory policy for foreign manufacturers, and a working standard is also considered for local manufacturers. The primary standard is checked online for shelf life and identity. Working standard is accepted if a valid certificate of purity, assay, identity, and loss on drying is available. Each analyst is expected to undertake analysis for three samples. However, the number of samples submitted for quality analysis may exceed the available analysts, and therefore, the timeframe from sample reception to result notification is 15 days to 3 months, which is considered acceptable.

**Figure 2:**
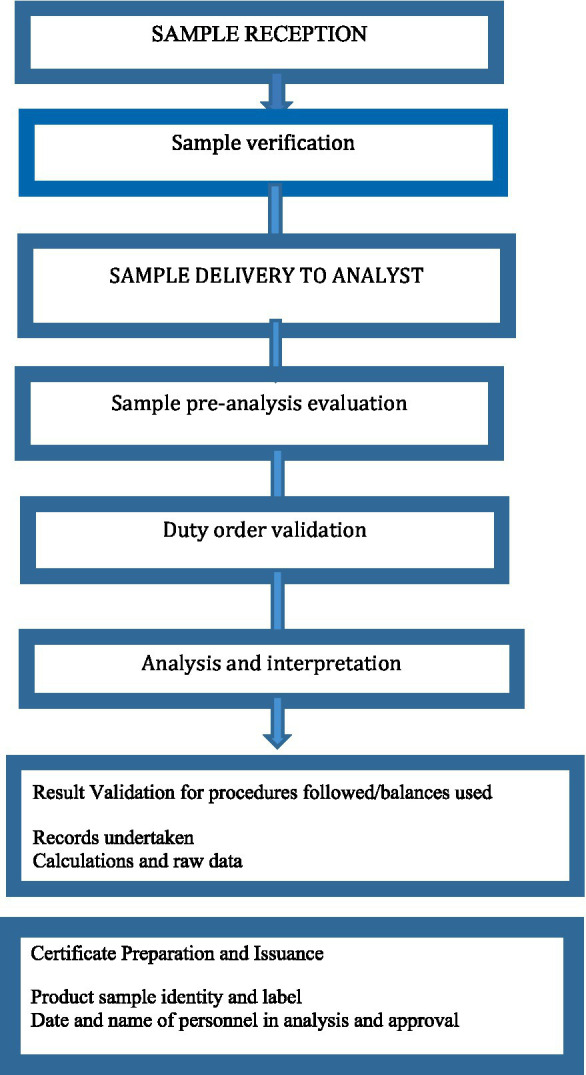
Sample processing flowchart in the sample quality assessment center in EFDA.

Reference literature for quality assessment result judgment considers USP, BP, and International Pharmaceuticals that are adopted in quality management documents. System suitability is also undertaken even during the use of pharmacopeia procedures before the real analytical procedure. If an in-house manufacturing method is to be used, the manufacturer must provide a method validation report, specifications for the finished product, and specific methods of analysis. During the issuance of results, the analyst, team leader, and director must sign the certificate of analysis (Figure 2). Conflict resolution procedures are also established when there is a grievance about the result notices. The quality assurance unit is responsible for grievance handling. When a grievance is reported, it will be rechecked by the same analyst and the case team.

### Mapping regulatory risks in product quality control: insights from fishbone diagram analysis

**Figure 3:**
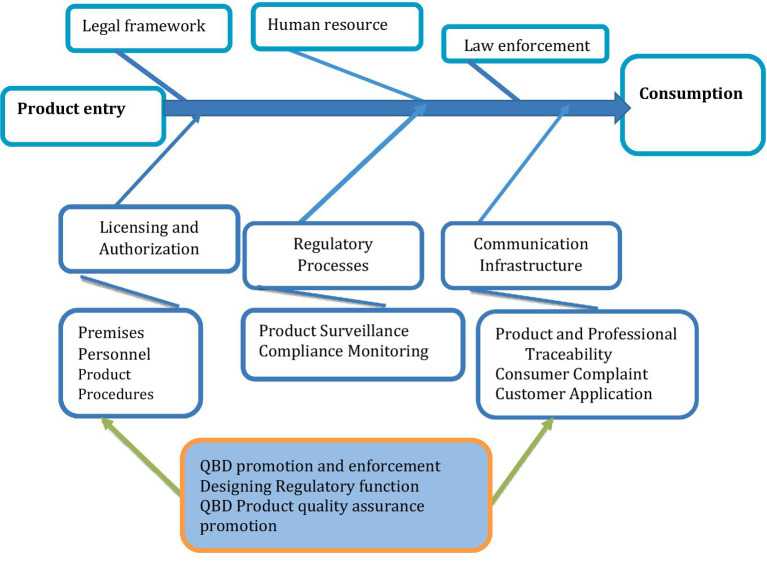
Fishbone diagram depicting regulatory processes for product quality assurance from product manufacture to consumption.

In the context of regulatory risk for product quality, the fishbone diagram can help pinpoint factors affecting the efficiency of the national product quality assurance system. It is used to identify and analyze the root causes of product quality problems and regulatory issues. The structure of a fishbone diagram for identifying risk factors in product quality regulation is summarized in Figure 3.

The ultimate objective of product quality regulation is to maintain the quality, safety, and efficacy of regulated products, ensuring that they do not have an undesired impact on social, economic, and public health, as well as the environment (44). Quality in this context refers to the fitness of a product or service for its intended purpose. For example, medicines for human consumption must be free from contaminants or impurities, contain active ingredients according to their labeled specifications, and exhibit the expected theoretical performance. Its proper drug quality can also be partly guaranteed for its safety and efficacy (45).

Products may have inherent safety concerns, and product quality regulation helps to maintain informed decision-making on their use through regulating the accompanying information for use. In essence, all product regulatory input and processes in product regulation need to be designed to minimize failures in maintaining quality, safety, and efficacy. Failures in product regulation are thus considered regulatory risk. The list of factors contributing to failure mode is derived from existing regulatory experience, and remedial actions are also discussed and recommended as risk management modalities. Regulatory risk is thus characterized by the probability of occurrence (O) of a failure mode and the severity of failure (S) in causing harm, combined with its probability of detection (D) of failure for corrective measures, giving a combination for regulatory risk evaluation (27).

Regulatory risk encompasses all processes and input parameters, together with their associated probabilities of occurrence, the potential harm of failure modes on public health, broader socioeconomic impacts, and the likelihood of detection failures within the regulatory system. Probability occurrence (O), risk severity (S), and ease of detection (D) are rated using five points each, with higher values representing greater risk (27). For example, higher rate values for detection indicate that the failure mode is very difficult to detect and understand, and lower values indicate a higher probability of detection. Probability and severity are rated with higher values when the failure mode has a higher probability of occurrence and the severity of the failure mode in causing regulatory failure is higher.

From a regulatory risk perspective, the study found that suboptimal performance (RPN = 80) and the circulation of substandard products (RPN = 75) were the critical failures related to a lack of premises and product compliance surveillance and monitoring, which were the major risk failures in the regulatory systems. A lack of transparency in the institutional licensing process (RPN = 64) was also identified as a major failure mode contributing to regulatory risks. The details of regulatory risk attributes and risk probability numbers were summarized in Supplementary file S2.

### Risk management and regulatory strengthening framework for National Pharmaceutical Regulatory Authority (NRAs)

Expert-based group discussions identified and prioritized key factors contributing to regulatory failure. This comprehensive risk failure analysis provides a structured approach to identifying and addressing key weaknesses within the pharmaceutical regulatory system (Supplementary file S2; Figure 2). Using a Failure Mode and Effects Analysis (FMEA) methodology, it evaluates risk attributes across regulatory segments based on occurrence, severity, and detectability. It calculates a risk priority number (RPN) to prioritize actions. The analysis reveals critical issues in legal frameworks, such as outdated proclamations and a lack of transparency, with corruption in law enforcement identified as a high-risk factor. Addressing this requires continuous legal review and implementation of digital information systems to foster guideline-based decision-making (46).

Human resource challenges, including inadequate staffing, low motivation, and skill gaps, were also highlighted. These call for improved recruitment guidelines, periodic institutional organogram reviews, and implementation of incentive packages and professional development plans. In the licensing and authorization segment, delays in feedback, lack of transparency, and application backlogs reflect systemic inefficiencies. These issues can be mitigated by implementing standard operating procedures (SOPs) for document auditing, application feedback, and dossier evaluation, as well as integrating online application tracking systems. The post-marketing surveillance and compliance monitoring areas emerged as the highest risk zones, with the circulation of substandard products and suboptimal regulatory performance (RPN = 80) posing significant public health risks. Solutions include adherence to pharmacovigilance guidelines, strengthened GMP enforcement, traceability systems, and focused SOPs for inspections and corrective actions (47). Quality control issues, such as measurement errors and inconsistency in reference standards, further highlight the need for robust analytical SOPs, validated reference materials, and digital testing systems. Communication infrastructure gaps, including the absence of online systems and incomplete regulatory websites, impede transparency and stakeholder engagement, necessitating investments in ICT and the creation of national databases for professionals and products.

Additionally, the lack of regulation in pharmaceutical promotions leads to the spread of misleading information, necessitating strict promotional guidelines and effective enforcement. Finally, the resistance to adopting Quality by Design (QbD) principles in regulation and manufacturing reflects institutional inertia, which can be addressed through capacity building and the promotion of model institutions. Furthermore, a risk-based framework provides actionable insights for strengthening NRAs, ensuring transparency, efficiency, and public health protection through systematic reform and targeted interventions (48).

### Strengths and limitations of the study

This study provides an in-depth analysis of the Ethiopian Pharmaceutical regulatory landscape using retrospective quality assessments, cross-sectional surveys, and expert-based interactive discussions. The use of convenience sampling for inclusion in a questionnaire survey and discussion using regulatory experts, wholesalers, and importers can be used for qualitative conclusions, such as the presence of defective products in the market, listing risk factors and mitigation strategies, and constraints existing in the regulatory setup. However, the use of this data cannot be extrapolated as a quantitative value representing a country profile, which rather needs a separate randomized study, which is a limitation of the current study. Additionally, risk factor analysis cannot be interpreted as it stands, as some factors are part of the other broader factors, such as corruption and suboptimal performance. Risk factor analysis from expert opinion is opinions from expert exposures and knowledge from experience. Besides this, regulatory risk factors should not be misinterpreted as direct causal factors for undesired findings. For example, non-compliance with regulatory law cannot be a direct cause for substandard product findings. Future longitudinal studies are rather suggested to explore more robust causal pathway associations and the impact of QBD-based reforms over time.

## Conclusion and future direction

Findings from this study revealed the presence of defective products in the market and constraints in the supply and regulation of medicinal products. Constraints in foreign currency, limitations in marketed product diversity and volume, as well as problems with the regulatory system, are indicated as existing challenges in the supply chain. Expert-based risk assessment has also outlined regulatory risk related to poor post-market surveillance, weak regulatory enforcement, and suboptimal performance (which can be due to corruption or other issues of workplace demotivation). Despite the current proportion of poor-quality products reflecting similar trends to those in global developing countries, the need to assess using broader representation remains important. Expert-based analysis of regulatory risks and management recommendations can help in developing a more efficient, transparent, and cost-effective quality assurance system in the pharmaceutical supply chain.

## Data Availability

The raw data supporting the conclusions of this article will be made available by the authors, without undue reservation.

## Footnotes

^1^ https://mrh.igad.int
