# Supplementary material for: Lessons from Ethiopian pharmaceutical regulation: a risk-based approach to overcome challenges and unlock opportunities
Source: Front Med (Lausanne). 2025 Sep 4;12:1484649. doi: 10.3389/fmed.2025.1484649 (PMC12444208; doi:10.3389/fmed.2025.1484649)
Supplement: Supplementary file 2 [file Table_2.DOCX]

**Supplementary File 2.** Regulatory Risk Attributes and Risk probability Number.

| **Regulatory segment** | **Risk Attribute** | **Failure Mode** | **O** | **S** | **D** | **RPN** | **Management prescription** |
| --- | --- | --- | --- | --- | --- | --- | --- |
| Legal Framework | Proclamations, Regulations/ Guidelines, Standard operating Procedures | Deficiency | 1 | 4 | 1 | 4 | Continuous and progressive review on proclamation and guidelines |
| Law enforcement | Decision | Lack of autonomy | 2 | 4 | 2 | 16 | Develop culture for guideline-based decision making |
|  | Transparency | Corruption | 3 | 4 | 4 | 48 | Information communication infrastructure Automation |
| Human resource  Recruitment | Competency | Low | 1 | 4 | 2 | 8 | Training/recruitment guidance |
|  | Background | Irrelevance | 2 | 4 | 2 | 16 | Recruitment guidance |
|  | Diversity and specialization | Low | 2 | 2 | 1 | 4 | Focused applicant Selection |
|  | Adequacy | Inadequate | 2 | 3 | 1 | 6 | Periodic institutional organogram review |
| Human Resource management | Motivation | Low | 2 | 4 | 1 | 8 | Promotion and incentive package guideline |
|  | Workload | Burden | 5 | 1 | 1 | 5 | Employment and |
|  | Skill development | Absence or deficiency | 1 | 3 | 1 | 3 | Continuous professional development plan |
| Licensing and authorisation | Application Process for registration | Transparency | 1 | 2 | 2 | 4 | Inclusion and review for standard operating procedures |
|  |  | Queuing failure | 1 | 3 | 1 | 3 | Online progress display |
|  |  | Inadequate document auditing and verification | 2 | 2 | 3 | 12 | SOP for document auditing |
|  | Application Content Review and feedback | Feedback Delay | 3 | 2 | 2 | 12 | Citizen charter for customer management |
|  |  | Incompleteness | 1 | 4 | 4 | 16 | Document checklist SOP  Feedback standard SOP |
|  |  | Absence of Timing record and review | 2 | 3 | 1 | 6 | Customer application and feedback timing record and review SOP |
|  |  | Backlogging | 4 | 2 | 1 | 8 | GMP guidance document |
|  | Dossier evaluation and review | Review and feedback delay |  |  |  |  | SOP for dossier review |
|  |  | Feedback incompleteness |  |  |  |  | SOP for review content and feed back |
|  |  | Backlogging |  |  |  |  | Adequate personnel Allocation |
|  | GMP | Poor GMP planning | 2 | 3 | 2 | 12 | GMP campaigning, reporting and feedback timing sop |
|  |  | Lack of transparency |  |  |  |  | GMP assessment checklist and SOP |
|  |  | Feedback timing and inconsistency | 1 | 4 | 2 | 8 | Response timeline guideline |
|  | Product Registration and marketing authorization Approval | Service Transparency | 2 | 3 | 3 | 18 | Service checklist SOP |
|  |  | Service fee inconsistency | 1 | 2 | 1 | 2 | Regulatory fee guideline |
|  |  | Absence of Timing record and review | 2 | 2 | 2 | 8 | Timing SOP |
|  | Institutional Licensing Process | Application Feedback Delay | 4 | 3 | 2 | 24 | Institutional standard guideline, service timing SOP, Use of online process monitoring |
|  |  | Timing record and review failure | 2 | 3 | 4 | 24 |  |
|  |  | Lack of Transparency | 4 | 4 | 4 | 64 |  |
| Premise, Product, professional and process Compliance Monitoring | Premises and Product Compliance surveillance and monitoring | Substandard product circulation | 3 | 5 | 5 | 75 | Adherence for PMS Guideline |
|  |  | Increased counterfeiting | 2 | 4 | 5 | 40 |  |
|  |  | Suboptimal performance | 4 | 4 | 5 | 80 |  |
|  | Professional practice monitoring | Non-professional engagement and illegal trade | 2 | 4 | 4 | 32 | SOP for monitoring and for administrative measures |
|  |  | Regulatory Incompliance | 2 | 3 | 4 | 24 | SOP for regulatory checklist  And procedures |
|  | Supply chain distribution procedures | Unsafe handling and management | 2 | 4 | 4 | 32 | SOP for GDP monitoring  SOP for corrective actions |
|  |  | Absence of recording and documentation | 4 | 3 | 4 | 48 | SOP for traceability monitoring |
|  |  | Absence of traceability | 3 | 4 | 4 | 48 | Traceability Guideline |
|  |  | Pricing unfairness | 2 | 1 | 3 | 6 | Pricing guideline |
|  | PMS Procedures | Sampling inconsistency | 3 | 4 | 4 | 48 | Sampling SOP |
|  |  | Recall failure | 4 | 4 | 2 | 32 | Recall SOP |
| Quality Control center | Sample Reception | Incomplete sample information | 3 | 3 | 2 | 18 | Guideline and SOP for sampling, sample processing, |
|  |  | In proper coding and labelling | 3 | 4 | 3 | 36 |  |
|  |  | Inadequate sample size | 2 | 3 | 5 | 18 |  |
|  | Quality Reference use | Inconsistency in reference guide | 2 | 2 | 2 | 8 | Establishing type of reference used for decision in quality manual |
|  |  | Reference standard quality | 3 | 4 | 4 | 48 | SOP for procurement, storage, use and disposal |
|  | Sample Analysis | Measurement errors | 3 | 4 | 4 | 48 | SOP for serial and parallel testing |
|  |  | Dilution errors | 2 | 4 | 4 | 32 | SOP for reading and estimation, Use digital measurement with minimal error |
|  | Sample result verification | Reading errors | 2 | 4 | 3 | 24 | SOP for decoding and reporting procedures |
|  | Sample result issuance | Delayed feedback delivery | 3 | 2 | 2 | 12 | Adherence to sample result SOP |
|  |  | Informal communication | 1 | 5 | 5 | 25 | SOP adherence for personal responsibility |
|  | Customer Feedback and Complaint submission | Failure to accept grievance | 2 | 3 | 2 | 12 | SOP for Grievance management |
|  |  | Lack of transparency | 2 | 3 | 3 | 18 | Complaint evaluation and corrective actions |
|  | Third party  Sample analysis | Absence of third-party verification | 2 | 2 | 3 | 12 | SOP for third part analysis and guideline |
|  |  | Absence of procedures | 2 | 2 | 3 | 12 | Clinical Trial |
| Communication infrastructure | Online Application | Absence of online system | 1 | 3 | 2 | 6 | Building online communication system among stake holders and end users,  Other modes of communication |
|  |  | Absence of online progressive feedback | 4 | 3 | 2 | 24 |  |
|  |  | Discontinuity | 4 | 3 | 2 | 24 |  |
|  | Product Traceability | Absence of product identification system | 3 | 4 | 4 | 48 | Medicine and device promotion control |
|  |  | Poor network accessibility | 4 | 3 | 3 | 36 |  |
|  | Professional Traceability | Absence of National database | 5 | 4 | 2 | 40 | Market Authorization/Product Registration |
|  |  | Absence of unique professional Identification | 3 | 4 | 2 | 24 | Establishing national database |
|  | Regulatory web site | Poor network accessibility | 4 | 4 | 2 | 32 | ICT development |
|  |  | Incomplete information display | 3 | 3 | 2 | 18 | Competent Website development |
|  |  | Lack of continuous Update | 3 | 3 | 3 | 27 | Website regular followup and update |
| Regulation for Pharmaceutical promotion | Product promotion | Misleading information to public | 3 | 4 | 5 | 60 | Promotion guideline and corrective measure implementation |
|  |  | Inadequate information | 3 | 4 | 2 | 24 |  |
| Building quality in system | Promotion for quality by design in product regulation | Resistance for new quality regulation concepts | 5 | 4 | 2 | 40 | Sector capacity building on quality be design paradigm |
|  | Promotion for QBD in product registration and marketing | Commitment to old regulatory procedures | 5 | 4 | 2 | 40 | Adoption of role model institution for QBD |
|  | Enforcement for quality by design Approaches in Product manufacturing | Failure to adopt new concepts for better efficiency in product assurance | 4 | 3 | 3 | 36 | Enforcement of QBD pharmaceutical product and institution registration |
